# Detonation Performance of Insensitive Nitrogen-Rich Nitroenamine Energetic Materials Predicted from First-Principles Reactive Molecular Dynamics Simulations

**DOI:** 10.1021/jacsau.4c00069

**Published:** 2024-03-21

**Authors:** Dezhou Guo, Yuanyuan Wei, Sergey V. Zybin, Yan Liu, Fenglei Huang, William A. Goddard

**Affiliations:** † State Key Laboratory of Explosion Science and Technology, Beijing Institute of Technology, Beijing 100081, People’s Republic of China; ‡ Materials and Process Simulation Center, California Institute of Technology, Pasadena, California 91125, United States

**Keywords:** Chapman−Jouguet, nitroenamine, ReaxFF, insensitivity, energetic performance

## Abstract

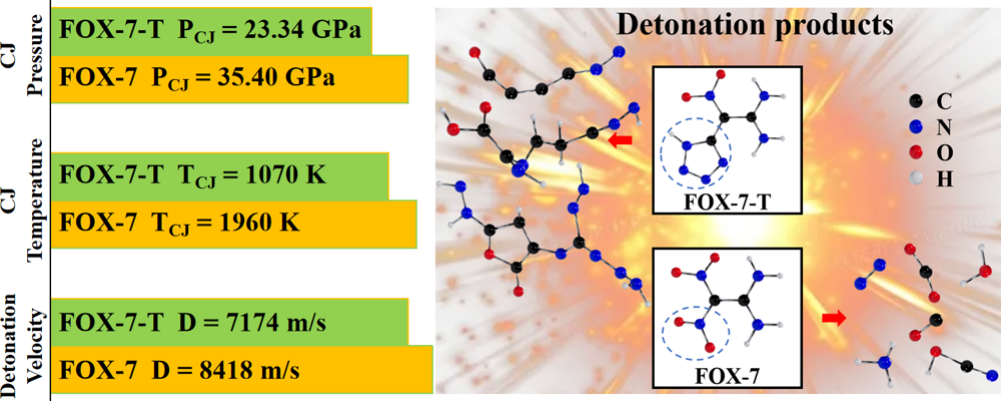

Because of the excellent combination of high detonation and low sensitivity properties of the 1,1-diamino-2,2-dinitroethylene (FOX-7) energetic material (EM), it is useful to explore new energetic derivatives that start with the FOX-7 structure. However, most such derivatives are highly sensitive, making them unsuitable for EM applications. An exception is the new nitroenamine EM, 1,1-diamino-2-tetrazole-2-nitroethene (FOX-7-T) (synthesized by replacing a nitro group with a tetrazole ring), which exhibits good stability. Unfortunately, FOX-7-T shows an unexpected much lower detonation performance than FOX-7, despite its higher nitrogen content. To achieve an atomistic understanding of the insensitivity and detonation performance of FOX-7 and FOX-7-T, we carried out reactive molecular dynamics (RxMD) using the ReaxFF reactive force field and combined quantum mechanics MD (QM-MD). We found that the functional group plays a significant role in the initial decomposition reaction. For FOX-7, the initial decomposition involves only simple hydrogen transfer reactions at high temperature, whereas for FOX-7-T, the initial reaction begins at much lower temperature with a tetrazole ring breaking to form N_2_, followed by many subsequent reactions. Our first-principles-based simulations predicted that FOX-7-T has 34% lower CJ pressure, 15% lower detonation velocity, and 45% lower CJ temperature than FOX-7. This is partly because a larger portion of the FOX-7-T mass gets trapped into condensed phase carbon clusters at the CJ point, suppressing generation of gaseous CO_2_ and N_2_ final products, leading to reduced energy delivery. Our findings suggest that the oxygen balance is an important factor to be considered in the design of the next generation of high-nitrogen-containing EMs.

## 1 Introduction

The great interest of high security in many engineering projects motivates continued efforts in design and synthesis of insensitive energetic materials (IEMs).^1−3^ An ideal representative IEM is 1,3,5-triamino-2,4,6-trinitrobenzene (TATB), which is widely used in applications requiring extreme safety and long-term storage, such as explosive formulations and low burning rate propellant components for space exploration.^4^ The low sensitivity to external stimuli of this “wood EM” comes from the high energy barrier of the initial C–N bond cleavage reaction and further decomposition processes that form large amounts of carbon clusters. These slowly decomposed clusters extend reaction time to hundreds or even thousands of nanoseconds.^5^ In contrast, typical conventional EMs, such as RDX and HMX, exhibit quite short reaction time of a few nanoseconds.^6,7^ Indeed, TATB is often regarded as a nonideal explosive because its complex detonation behaviors cannot be well described by classical Chapman–Jouguet (CJ) chemical equilibrium theory.^8−10^ Actually, incomplete carbon oxidation limits the TATB energy release, resulting in a low detonation velocity of ∼7500 m/s detonation and a low detonation pressure of ∼30 GPa.^11,12^

Recently, the single crystal of 1,1-diamino-2,2-dinitroethene (known as FOX-7 or DADNE) was shown to be strongly insensitive to shock wave initiation.^13,14^ Based on recent experimental profile measurements and time-resolved Raman spectra, no onset of any chemical reaction was observed with the shock-wave-compressed single crystal to ∼20 GPa.^13^ A very recent work even extended its shock insensitivity to as high as 25 GPa.^6^ Thus, FOX-7 belongs to the group of IEM because the onset of chemical decomposition for commonly used EM of PETN or RDX crystal is around 5–6 GPa.^15,16^ Surprisingly, this work found that the FOX-7 detonation performance is well described by classical CJ theory, which is opposite the observations of other common IEMs.^6,17^ Compared with the long reaction zone of TATB, the instantaneous chemical reactions of FOX-7 are completed within 0.7 ns after the detonation front passes through the crystal.^6^ This encouraged us to study the distribution of detonation products at the CJ state to understand the detonation performance of FOX-7.^18^

Inspired by FOX-7, a number of energetic derivatives have been generated based on the nitroenamine structure, such as 1-amino-1-hydrazino-2,2-dinitroethene^19^ and 1-amino-1-hydroxyamino-2,2-dinitroethene.^20^ However, thermal instability limits their further applications. Very recently, Tang et al.^21^ designed a series of nitrogen-rich FOX-7-like compounds, in which 1,1-diamino-2-tetrazole-2-nitroethene (denoted as FOX-7-T here) was synthesized by replacing one nitro group with a heterocyclic tetrazole ring. FOX-7-T shows promising insensitivity to external stimuli such as heat and impact. This good sensitivity of FOX-7-T was speculated to arise from its planar molecule structure and parallel intermolecular packing. Another important factor for stable thermal stability could be the slow initial decomposition reaction, but the reaction mechanism has remained unknown. In addition, based on the empirical EXPLO5 program, the predicted detonation velocity and detonation pressure for FOX-7 are 8613 m/s and 31.6 GPa, respectively, at an ambient density of 1.845 g/cm^3^. These are much better than the predicted detonation velocity of 8499 m/s and the detonation pressure of 26.7 GPa for FOX-7-T at an unrealistically high density of 1.83 g/cm^3^ (the experimental density at ambient conditions^21^ is 1.69 g/cm^3^ and the calculated density using the Keshavarz equation implemented in the new RoseBoom code^22^ is 1.659 g/cm^3^). Therefore, the detonation performance of FOX-7-T under ambient conditions is expected to be much lower than that of FOX-7. This contradiction that the higher nitrogen content FOX-7-T has a lower detonation performance than that of FOX-7 cannot be explained by the EXPLO5 program, probably because the empirical BKW/JCZ3-fitted equation of the detonated state does not provide the essential chemical and physical details during the detonation processes. Moreover, EXPLO5 indicates nothing about the distribution of detonation products at the CJ State. This makes it important to study the underlying atomistic mechanisms of FOX-7-T at the CJ state based on first-principles-based methods.

In this paper, we determined the initial decomposition reaction mechanisms using quantum mechanics molecular dynamics (QM-MD) cook-off simulations on the FOX-7 and FOX-7-T systems as a function of temperature from 300 to 3000 K. To predict the detonation parameters and products at the CJ state, we applied a practical method combining ReaxFF MD (RxMD) and QM-MD. RxMDs are first applied to achieve the long convergence process from an inert material to the equilibrated detonated state. Then, QM-MDs are performed to correct the nonbond atomic interactions from RxMD and to describe the statistical pressure value accurately. The predicted CJ state parameters provide the means to quantify the detonation performance of FOX-7 and FOX-7-T. Herein, our first-principles-based simulations describe the full sequence of complex reactive processes involved in product formation beginning from the initial decomposition to the final hot dense CJ state, with no *ad hoc* assumptions for the reaction processes or product compositions. Thus, our simulations provide a detailed atomistic description of the detonation properties and final products for the FOX-7 and FOX-7-T systems.

## 2 Methods

### 2.1 Cell Optimization Details

The unit cell structures of FOX-7 and FOX-7-T were taken from the Cambridge Structural Database. We optimized these structures using the PBE-D3 flavor of density functional theory (DFT) implemented in the VASP package with a plane-wave basis set.^23,24^

The optimized cell parameters for FOX-7 at 300 K are *a* = 6.82 Å, *b* = 6.88 Å, *c* = 11.32 Å, α = 90°, β = 90.91°, and γ = 90° from the DFT simulations, leading to a density of 1.85 g/cm^3^. The DFT-optimized cell parameters agree very well with the X-ray experimental values of *a* = 6.94 Å, *b* = 6.57 Å, *c* = 11.32 Å, α = 90°, β= 90.55°, and γ = 90°, leading to a density of 1.91 g/cm^3^ at 173 K, as shown in Table 1. The optimized cell parameters for FOX-7-T are *a* = 4.33 Å, *b* = 13.85 Å, *c* = 16.93 Å, α = 104.73°, β = 91.78°, and γ = 85.73° (at 300 K), leading to a density of 1.68 g/cm^3^, in reasonable agreement with *a* = 4.98 Å, *b* = 13.11 Å, *c* = 16.93 Å, α = 101.61°, β = 91.18°, and γ = 90.05° (at 293 K) from X-ray experiments with a density of 1.69 g/cm^3^. Thus, the PBE-D3 flavor of DFT describes the crystal structure of both FOX-7 and FOX-7-T well.

**Table 1:** Cell Parameters from DFT Simulations and Experiments for FOX-7 and FOX-7-T

	*ρ* (g/cm^3^)		*a* (Å)		*b* (Å)		*c* (Å)		α (°)		β (°)		γ (°)	
	DFT	exp.	DFT	exp.	DFT	exp.	DFT	exp.	DFT	exp.	DFT	exp.	DFT	exp.
FOX-7	1.85	1.91	6.82	6.94	6.88	6.57	11.32	11.32	90	90	90.91	90.55	90	90
FOX-7-T	1.68	1.69	4.33	4.98	13.85	13.11	16.93	16.93	104.73	101.61	91.78	91.18	85.73	90.05

The QM structure optimization and equilibrium QM-MD were predicted using the Perdew–Burke–Ernzerhof (PBE) Generalized Gradient Approximation (GGA) functional of DFT including using the Grimme D3 empirical van der Waals interactions using Becke–Johnson damping parameters, as implemented in VASP.^25,26^ We applied the projector augmented-wave (PAW) method for the exchange–correlation interaction and the core–valence interactions.^27^ To determine the electron partial occupancies, we applied the tetrahedron method with Blöchl corrections.^28^ The kinetic energy cutoff for the plane wave expansions was set to 500 eV. The convergence criteria for energy and force were set to 1 × 10^–6^ eV and 1 × 10^–4^ eV Å^–1^ for the electronic self-consistent field (SCF) procedure and ionic relaxation loop, respectively. The Brillouin zone integration for QM-MD was performed on Γ-centered symmetry-reduced Monkhorst–Pack meshes with a 1 × 1 × 1 *k*-point grid mesh. A time step of 1.0 fs was used for integrating the equation of motion in the QM-MD simulations.

### 2.2 QM-MD Simulations of the Initial Steps of the Reactions

We used the Born–Oppenheimer QM-MD approach implemented in the VASP package to examine the initial decomposition reactions during cook-off simulations. We first heated the cells at a constant rate from 10 to 300 K within 2 ps. Then, the systems were equilibrated at 300 K for 2 ps using the *NVT* ensemble (constant volume, constant temperature, and constant number of atoms) using the Nose–Hoover thermostat. Finally, the system was heated from 300 to 3000 K at a constant heating rate of over 20 ps.

We applied a molecular fragment recognition analysis algorithm with the connectivity matrix and bond orders at 0.1 ps intervals to analyze the fragments of final products.^29^ Under high-pressure conditions at the CJ state (20–40 GPa), some final products of EMs tend to form clusters instead of gases. To appropriately assess the products, we used the cutoffs as in Table 2. Independent molecules were identified through bond orders compared to the cutoff values. Each such molecule was then assigned a particular ID number to track the reaction pathways. We set up a time window of 1.0 ps to avoid artificial breaking and formation of bonds induced by thermal fluctuations. The bond orders were computed using ReaxFF by converting the QM-MD trajectory to the MD trajectory.^30^

**Table 2:** Bond Order Cutoff Values for Various Atom Pairs

	C	H	N	O
C	0.55	0.40	0.30	0.80
H		0.55	0.55	0.40
N			0.45	0.55
O				0.65

### 2.3 Simulation Method to Predict the CJ State

The Zeldovich-von Neumann-Döring (ZND) detonation model describes the detonation wave propagating in a reactive medium material, leading to an exothermic reaction zone behind the shock front wave.^31^ The CJ state refers to the specific hot dense state at the end of the reaction zone of a sustained detonation wave, during which the chemical reactions achieve dynamic equilibration. According to the conservation law of mass and momentum before and after a detonation wave, the Hugoniot equation of the material can be expressed as
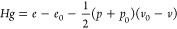
1where *p* is the pressure, *e* is the specific internal energy, and *v* is the specific volume. Herein, the term “specific” refers to the quantity per unit mass, and the subscript “0” refers to the quantity in the initial unshocked state.

This set of *e*, *v, and p* parameters can be expressed as a function of two independent variables of *v* and *T. H* = 0 expresses the conservation of energy before and after the detonation shock wave, linking *v* and *T* to a mathematical solution. To predict the *e*_0_, *p*_0_, and *v*_0_ parameters of the initial state, we equilibrated the system at 300 K for 20 ps with *NVT* QM-MD and averaged the values of the last 10 ps. For the thermodynamic properties of the detonated states, we first performed long RxMD simulations to describe the whole chemical dissociation process from the inert initial crystal to final detonated products, followed by QM-MD simulations for 20 ps to describe the final detonated state and the nonbond interactions accurately. The pressure and total energy of the complete decomposition state were obtained from the QM-MD simulations over the last 10 ps.

On the *P-ρ* (pressure–density) plane, the fully detonated Hugoniot curve (*H* = 0 curve) collects all possible states of fully detonated products. Here, we fit five *H* = 0 points into a quadratic polynomial to accurately describe the Hugoniot curve. These *H* = 0 points were obtained by carrying out 25 *NVT* calculations from five different sets of temperatures with five different densities for each temperature.

The CJ point is the tangent point between the quadratic polynomial Hugoniot curve and the linear polynomial Rayleigh line (which is a straight line that connects the points related to the initial and final states on the *P–V* plane). We can then obtain the location of the CJ state from these two curves in the *P–V* plane. Finally, we calculated the detonation velocity (*D*_CJ_) from the CJ pressure (*P*_CJ_) and predicted the CJ temperature (*T*_CJ_) using a quadratic polynomial fitted to the *T-V*/*V*_0_ curve.

Oxygen balance is an index of the deficiency or excess of oxygen in a compound required to convert all C into CO_2_ and all H into H_2_O.^32^ It is an important parameter to evaluate the degree of oxidization of energetic materials during detonation.^33^ The formula for oxygen balance for C_*a*_H_*b*_N_*c*_O_*d*_ can be expressed as
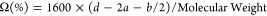
2

## 3 Results and Discussion

### 3.1 Reaction Mechanisms at High Temperatures from QM-MD Simulations

**Figure 1:**
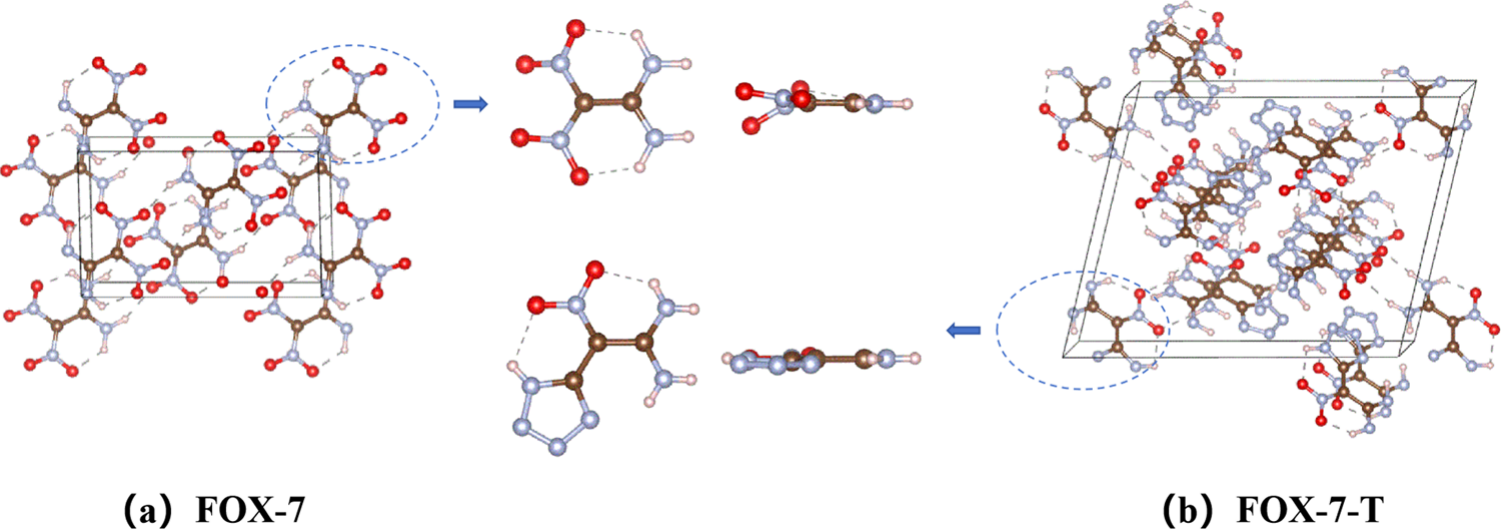
Structures for the unit cell and single molecule of (a) FOX-7 and (b) FOX-7-T. The C, N, H, and O atoms are represented by brown, light blue, white, and red balls, respectively.

We start with the unit cell of FOX-7 containing four molecules (56 atoms) and the unit cell of FOX-7-T containing six molecules (102 atoms), as shown in Figure 1.

**Figure 2:**
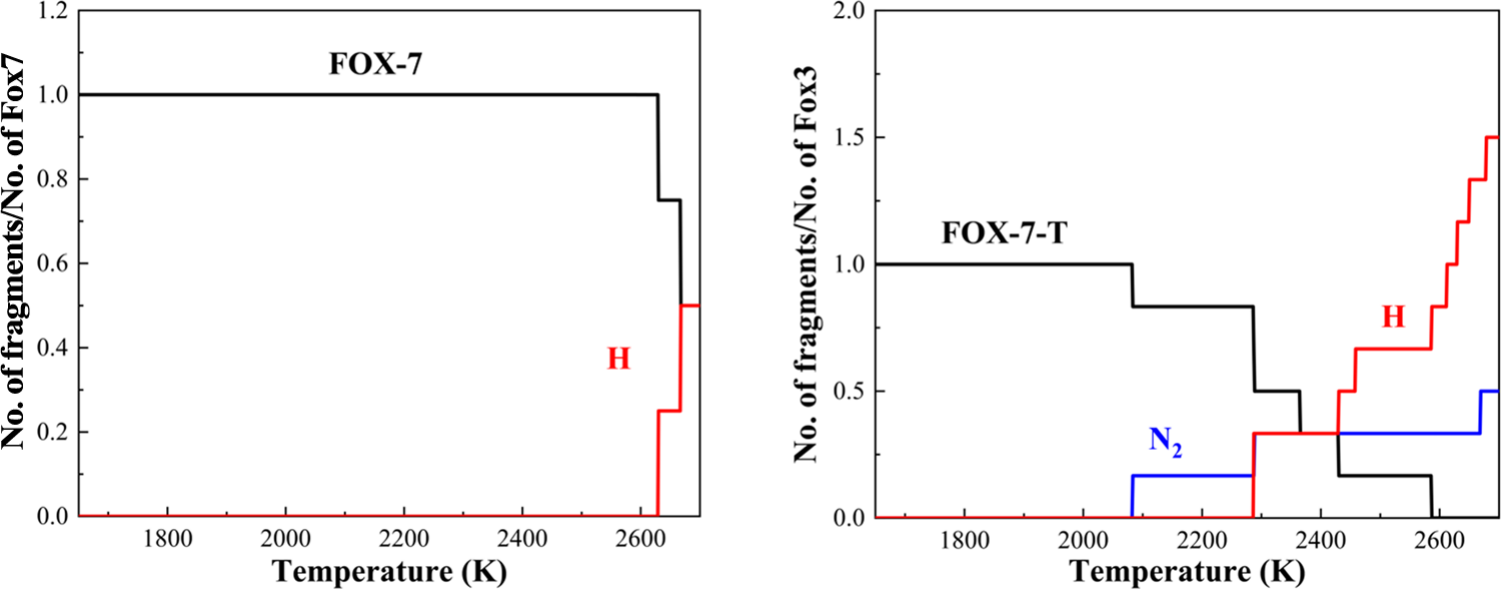
Species analysis for the decomposition of FOX-7 and FOX-7-T heated from 300 to 3000 K over 20 ps.

In order to examine the thermal stability and to understand the initial decomposition reaction mechanisms of FOX-7 and FOX-7-T, we analyzed the molecular fragments during the cook-off simulations from 300 to 3000 K, as plotted in Figure 2.

**Figure 3:**
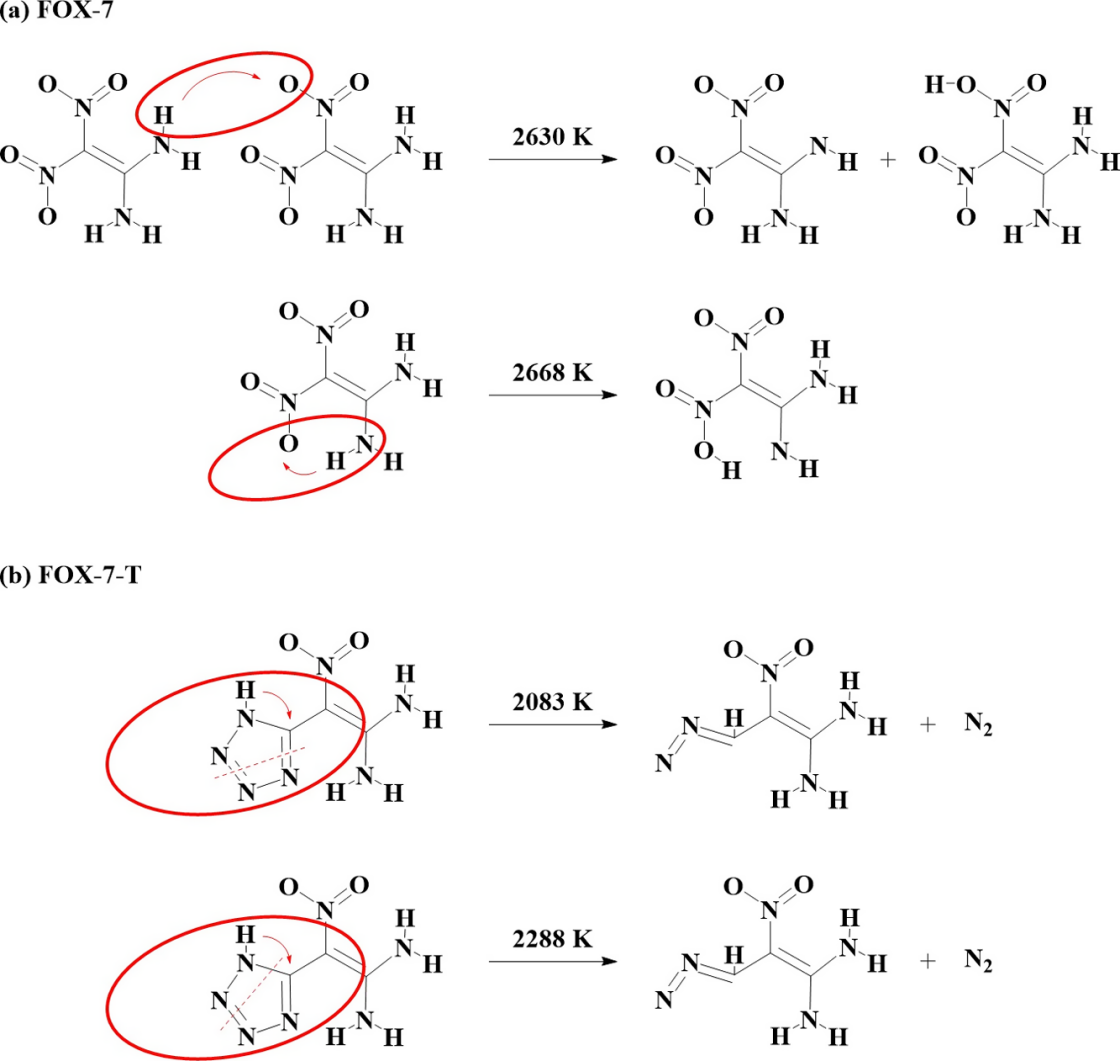
Initial decomposition reactions for (a) FOX-7 and (b) FOX-7-T.

For FOX-7, no reactions were observed until ∼2600 K. Its excellent thermal stability comes from the molecular unique “push-pull” electronic delocalization, strong hydrogen bonding, and laminated molecule layer arrangement.^34−39^ We observed an intermolecular proton transfer between an -NH_2_ group and a nearby -NO_2_ group at 2630 K and an intramolecular proton transfer between an -NH_2_ group and a nearby -NO_2_ group at 2668 K, showing that hydrogen transfer is the first reaction step in the initial thermal decomposition reactions, as shown in Figure 3a. Our results agree with previous ab initio molecular dynamics simulations that the inter- and intramolecular hydrogen transfers are the important initial decomposition pathways of FOX-7.^40^

For FOX-7-T, we found much earlier and more complex initial reactions during the heating process. First, molecular decomposition starts with C–N and N–N bonds breaking within the tetrazole ring, leading to N_2_ dissociation at 2083 K. At the same time, the isolated proton from the tetrazole was captured by the C radical of the ring, as shown in Figure 3b. A similar N_2_ dissociation and molecule rearrangement reaction was observed again at 2288 K. As the temperature was increased continuously, proton transfers between the -NH_2_ group and the -NO_2_ group were observed frequently, indicating that the N_2_ dissociation accelerates further molecular decomposition.

Thus, the ring-breaking step is the key reaction mechanism in the initial thermal decomposition of FOX-7-T, providing the starting point for subsequent decomposition reactions. Although showing a lower stability than that of FOX-7, the FOX-7-T system is more stable than many systems we studied previously, such as BCHMX^41^ (*T*_dec_ = 1700 K), TKX-50^42^ (*T*_dec_ = 1700 K), 4,4′-bis(dinitromethyl)-3,3′-azofurazanate MOF^43^ (*T*_dec_ = 1970 K), MTO^44^ (*T*_dec_ = 1800 K), and MTO_3_N^44^ (*T*_dec_ = 2000 K).

Thus, the strategy of substituting a nitro group with a high nitrogen content ring to form a planar molecular structure remains a promising means of improving the stability of nitroenamine EMs.

### 3.2 CJ State Prediction

**Figure 4:**
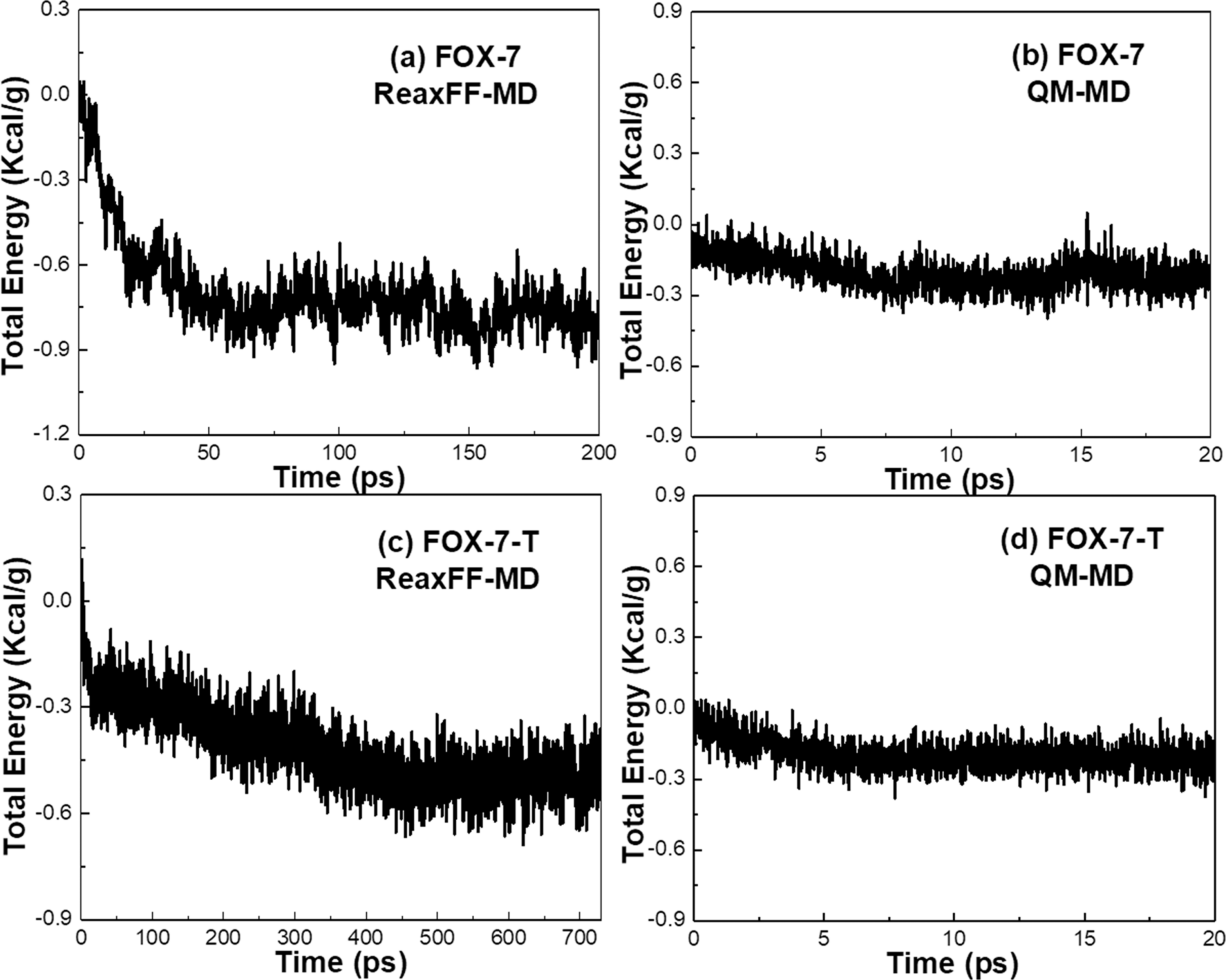
Time evolution of the total energy per unit mass in the cook-off simulation. The initial energy is set to zero as a reference. (a) ReaxFF-MD for the first 200 ps and (b) QM-MD for the last 20 ps at *T* = 2200 K and *V*/*V*_0_ = 0.65 for the FOX-7 system. (c) ReaxFF-MD for the first 730 ps and (d) QM-MD for the last 20 ps at *T* = 1400 K and *V*/*V*_0_ = 0.65 for the FOX-7-T system.

The CJ state is a point in the detonated Hugoniot curve of the EM in the *P–V* plane. We carried out a series of 25 long cook-off simulations using ReaxFF with prescribed volume compressions and held the system at appropriate temperatures to achieve completely reacted states. Five sets of temperatures were set, and five different densities were considered for each temperature. The range of these combinations of temperatures and volumes was tested initially to bracket final Hugoniot values close to zero. We found that ∼200 ps RMD for the FOX-7 system and ∼730 ps for FOX-7-T were sufficient to reach the final equilibrated detonated state. Figure 4a shows the total energy evolution of FOX-7 at *V*/*V*_0_ = 0.65 with *T* = 2200 K, and Figure 4c shows the energy of FOX-7-T at *V*/*V*_0_ = 0.65 with *T* = 1400 K.

For the FOX-7 system, an initial 25 ps of exponentially decreasing energy was observed due to the dramatic exothermic reactions. Next, 50 ps with mildly decreasing energy was observed due to the system gradually reaching the equilibrium state, which was verified by the subsequent 100 ps in which the energy remained unchanged. For the FOX-7-T system, a similar initial 25 ps fast decomposition period was observed, but this was followed with a 450 ps linear decreasing energy before convergence (due to the low external temperature). We conducted an extra 200 ps to confirm that the system had reached equilibrium.

Finally, we extracted the atom positions and velocities at the end of RMD simulations (200 ps for FOX-7 and 730 ps for FOX-7-T) and used these data as input for QM-MD simulations for another 20 ps to obtain an accurate first-principles description of the product states. These systems have reached equilibrium since the total energy remains constant for the last 20 ps as shown in Figure 4b,d. The properties of fully decomposed states, such as Hugoniot value, pressure, and total energy, were determined by averaging the last 10 ps QM-MD simulations. The fluctuations of energy and pressure during RMD and QM-MD simulations come from the high temperature. The equation of pressure and kinetic energy are shown in the Supporting Information.

**Figure 5:**
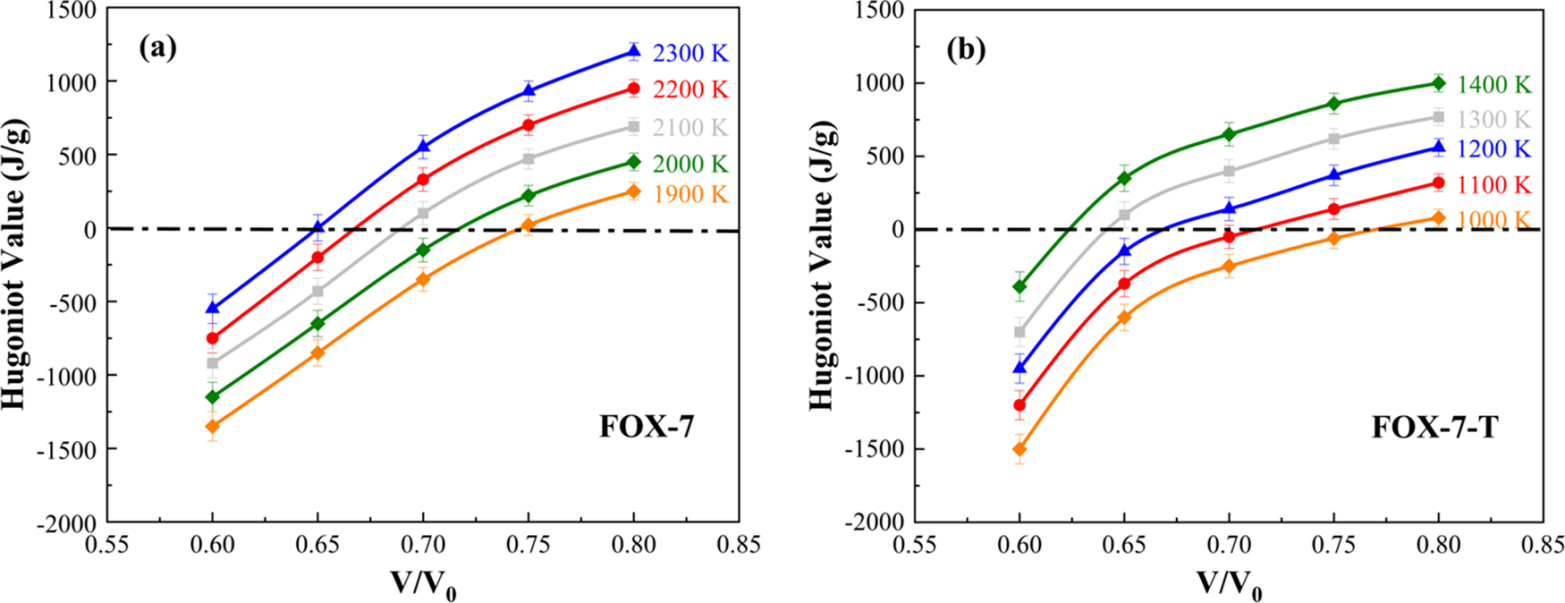
Spline fitted isotherms of the Hugoniot values and the volume compression ratios for five sets of temperatures of (a) FOX-7 and (b) FOX-7-T. The intersections of isotherms with the *Hg* = 0 line (the dotted–dashed line) provide the detonated states on the fully reacted Hugoniot curve.

**Figure 6:**
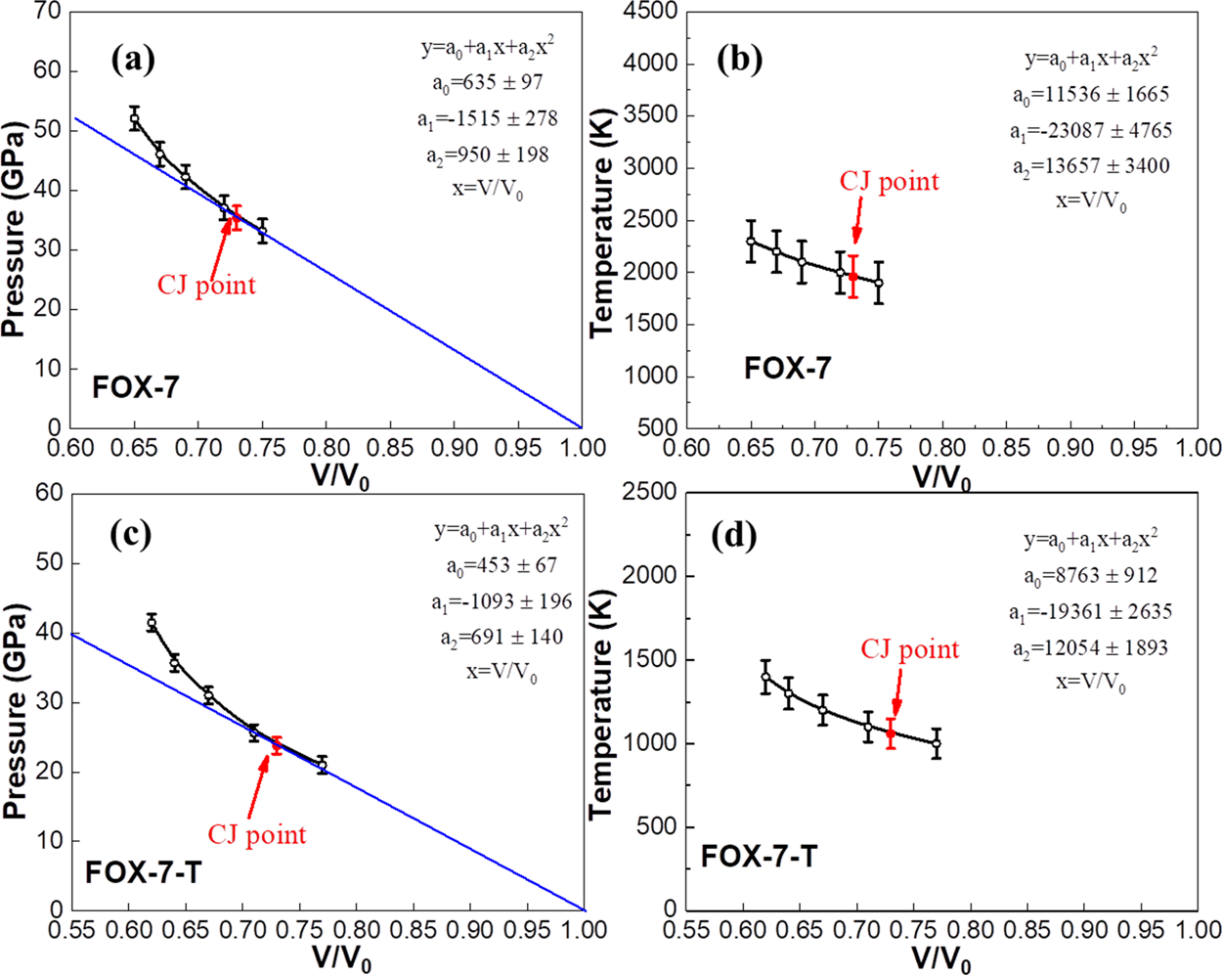
Hugoniot curve of the fully reacted state and the predicted CJ point for (a) FOX-7 and (c) FOX-7-T. The quadratic polynomial-fitted temperature–volume compression ratio curve for (b) FOX-7 and (d) FOX-7-T. The CJ points marked as red dots are determined by the tangent point of the Rayleigh line and the fully reacted Hugoniot curve.

For each specific temperature, we obtained a family of Hugoniot values by changing the volume compression ratio, followed by spline fitting to obtain the isotherm. Five isotherms coming from five temperatures with specific volume compression ratios are shown in Figure 5. Since *Hg* = 0 represents the energy conservation before and after the shock wave, the intersections of the isotherms with the *Hg* = 0 axis locate the volume compression ratios of the fully reacted Hugoniot curve. Therefore, five sets of thermodynamic parameters (*V*/*V*_0_, *T*) of the detonated states were found, and the detonation pressure was determined by the final products at these volume and temperature conditions. We used a quadratic polynomial to fit these five pressures in the *P*-*V*/*V*_0_ plane to predict the detonation Hugoniot curve that describes the equation of state of the products at the end of the reaction zone, as shown in Figure 6a for FOX-7 and Figure 6c for FOX-7-T. The CJ point is determined by the tangent point between the Hugoniot curve and the Rayleigh line, represented by the red dots in Figure 6a,c. We also fitted the temperature and volume into a quadratic polynomial from which the CJ temperature was obtained, as shown in Figure 6b, d. Thus, the detailed detonation properties were quantified by the parameters of the CJ state.

The predicted detonation properties for FOX-7 and FOX-7-T are listed in Table 3. For FOX-7, the predicted detonation pressure from our simulation is *P*_CJ_ = 35.40 ± 2.01 GPa at an initial density *ρ*_0_ = 1.85 g/cm^3^, agreeing very well with the experimental data of ∼35 GPa,^6^ as shown in Table 4. The predicted detonation velocity is *D*_CJ_ = 8.418 km/s, which compares well with the experimental data of 8.335 km/s at a density of 1.76 g/cm^3^, 8.000 km/s at a density of 1.7 g/cm^3^, 8.325 km/s at adensity of 1.78 g/cm^3^, and 8.300 km/s at a density of 1.79 g/cm^3^.^44−47^ Thus, our results are in good agreement with experimental data, validating our results.

**Table 3:** Detonation Properties at the CJ State for FOX-7 and FOX-7-T

	FOX-7		FOX-7-T	
	RxMD&QMMD	EXPLO5^21^	RxMD&QMMD	EXPLO5^21^
Density (g/cm^3^)	1.85	1.845	1.68	1.83^a^
*P*_CJ_ (GPa)	35.40 ± 2.01	31.6	23.34 ± 1.14	26.7
*T*_CJ_ (K)	1960 ± 213		1070 ± 95	
*D*_CJ_ (km/s)	8.418 ± 2.42	8.613	7.174 ± 1.78	8.499

a FOX-7-T was artificially compressed to a density close to FOX-7 to compare the detonation performance.

**Table 4:** Detonation Properties Estimated Previously for FOX-7 (Reported as Experimental)^6,45−48^

Density (g/cm^3^)	1.76^45^	1.7^46^	1.78^47^	1.79^48^	unknown^6^^,^^a^
*D*_CJ_ (km/s)	8.335 ± 0.025^b^	8.0^c^	8.325 ± 0.08^d^	8.3^e^	unknown^a^
*P*_CJ_ (GPa)	27.9	26	28.4	28	35^f^

a Neither density nor detonation velocity was mentioned.

b This detonation velocity was obtained by measuring the arrival time to piezo-pin transducers along the cylinder axis in cylinder tests with a density of 1.76 g/cm^3^ (consisting of 98.5 wt % of FOX-7 and 1.5 wt % of wax).

c This detonation velocity was measured through compression probes in the cylinder tests.

d This detonation velocity was determined by the method of short-circuit sensors in which time intervals were measured at three distances in a charge of 20 mm diameter. The velocity was obtained as a ratio between the distance and the corresponding time interval.

e This detonation velocity was measured through three short-circuit sensors in the water test with a density of 1.79 g/cm^3^ (consisting of 94 wt % of FOX-7 and 6 wt % of Viton A).

f This CJ pressure was determined through the extrapolation of sound speeds determined using the EOS of the detonation products and the *U*_s_ – *u*_p_ Hugoniot curve for the detonation products for shock compressed FOX-7.

For FOX-7-T, we predicted the detonation velocity *D*_CJ_ = 7.174 ± 1.78 km/s and *P*_CJ_ = 23.34 ± 1.14 GPa at an initial density *ρ*_0_ = 1.68 g/cm^3^. There are no experimental data available for FOX-7-T, but we expect these results to have similar accuracy as FOX-7.

For FOX-7-T, the CJ temperature is *T*_CJ_ = 1070 ± 95 K, which is 45.4% lower than the *T*_CJ_ = 1960 ± 213 K for FOX-7. Since the nitrogen percentage per unit mass for FOX-7-T is 19.47% higher than that of FOX-7, we expect that FOX-7-T would produce more nitrogen gases during detonation so that this low energy release of FOX-7-T comes from the significant amount of unoxidized carbon and hydrogen atoms due to the low oxygen balance of −60.82%. The CJ pressure for the FOX-7-T system is 34.1% lower than that of FOX-7, leading to a 15.1% lower detonation velocity of FOX-7-T compared to that of FOX-7. We expect that this low external energy delivery capability of FOX-7-T comes from the existence of far more condensed phase aggregates in the detonated system. This weakened detonation performances of FOX-7-T agrees with the prediction from the EXPLO5 program that even an 8% volume compression shows a lower detonation velocity and detonation pressure compared with those of FOX-7 at the ambient condition.^21^

FOX-7-T has a molecular structure similar to FOX-7 with only a functional group difference, but FOX-7-T shows dramatically weakened detonation properties. We attribute this to the low oxygen balance, leading to excess unoxidized detonation products compared with those of FOX-7. Thus, we analyzed the detonated product distributions at the CJ states with the fragment analysis program by averaging the last 10 ps QMD simulations, as shown in Table 5.

**Table 5:** Detonation Products Predicted at the CJ State and after Relaxation to Ambient Pressure for FOX-7 and FOX-7-T

		FOX-7		FOX-7-T	
		CJ	Expanded	CJ	Expanded
Density (g/cm^3^)		1.85		1.68	
Main products (mol/mol)	N_2_	1.50 ± 0.00	1.50	2.00 ± 0.00	1.83
	CO_2_	0.50 ± 0.14	1.00		0.33
	H^+^	2.19 ± 0.55		0.07 ± 0.02	
	H_2_				0.50
	CO		0.50		0.33
	H_2_O	0.50 ± 0.12	1.00	0.34 ± 0.02	0.17
	HO^–^	0.31 ± 0.08			
	NH_3_	0.25 ± 0.00	0.50		0.33
	N_2_H_2_				0.33
	CHO_2_	0.11 ± 0.03			
	CHON		0.5		
Other molecules (g/g)					
Carbon clusters		0.59	none	0.64	0.44
Composition		C_2.89_H_3.30_N_1.23_O_5.46_		C_4.78_H_6.77_N_4.78_O_2.64_	C_4.67_H_4.00_N_4.67_O_1.67_
Carbon cluster material recovery (%)					
C		88.28		100.00	77.78
H		50.39		84.90	40.00
N		18.75		42.86	33.33
O		83.20		82.84	41.67

**Figure 7:**
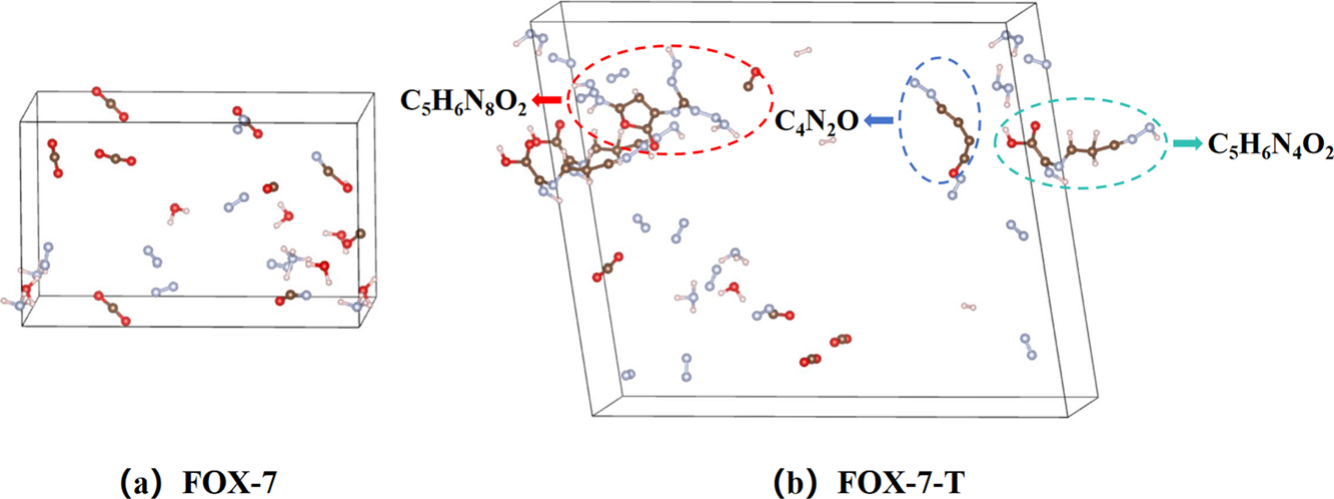
Detonation products after relaxation to ambient pressure for (a) FOX-7 and (b) FOX-7-T. For FOX-7, the main products are N_2_, CO_2_, H_2_O, CO, NH_3_, and CHON, with no remaining carbon clusters. For FOX-7-T, a large portion of carbon clusters remain in the condensed phase, suppressing the generation of gaseous products.

For FOX-7 in the CJ state, N_2_, CO_2_, H^+^, H_2_O, HO^–^, and NH_3_ are the dominant detonation products. In addition, 0.59 g of carbon clusters per gram of FOX-7 was produced with the atoms remaining aggregated in clusters that are 88.28% C, 50.39% H, 18.75% N, and 83.20% O. We expect that these clusters with an average formula go into the gas phase in the 8-fold expansion of C_2.89_H_3.30_N_1.23_O_5.46_ would decompose into the gas products during isoentropic expansion. In order to verify this, we applied a “linear volume expansion”^31^ with variable-volume RxMD simulations: the system volume *V*_0_ was expanded slowly at a linear rate until reaching 8*V*_0_ at 20 ps. Then, we obtained a first-principles description through a 1 ps QM-MD simulation starting with atom positions and velocities in the last step of the RxMD simulations. This procedure leads to an internal pressure value to ∼1 atm, allowing for a direct comparison to the detonation products in calorimetric experiments in which detonated products are expanded from the CJ point to normal pressure, as shown in Table 5. As expected, we found that all of these agglomerates decompose easily into final gas-phase products with no remaining carbon clusters, as shown in Figure 7. This explains that although the explosion of FOX-7 is hard to trigger, its fast decomposition in the reaction zone arises without the stubborn carbon cluster formation observed in ammonium nitrate shocks.

For FOX-7-T, we found that N_2_, H_2_O, and H^+^ are the dominant detonation products. However, compared with the FOX-7 system, neither CO_2_ nor CO was formed in FOX-7-T, indicating that all C atoms are trapped into aggregates. 0.64 g carbon clusters per gram of the FOX-7-T molecule were produced with an overall composition of C_4.78_H_6.77_N_4.78_O_2.64_, trapping 100.00% of C, 84.90% of H, 42.86% of N, and 82.84% of the O atoms at the CJ state. After the FOX-7-T system pressure was reduced to ∼1 atm, we found that many clusters remained in the condensed phase instead of going into the gas phase with 77.78% of C, 40.00% of H, 33.33% of N, and 41.67% of O atoms still trapped in clusters with an overall formulation of C_4.67_H_4.00_N_4.67_O_1.67_. Formation of these liquids or solids suppresses the generation of gaseous products, leading to a much lower detonation pressure and a much lower detonation velocity. These solids arise from the lack of oxygen to oxidize carbon, which also traps many nitrogen atoms inside clusters, leading to much fewer N_2_ gases being produced and less energy being released. Thus, a very low detonation temperature is achieved for FOX-7-T.

## 4 Conclusions

In summary, we applied first-principles-based reactive MD simulations by combining ReaxFF-MD and QM-MD to predict the initial reaction mechanisms and detonation performance of FOX-7 and FOX-7-T. The key points from our simulations are as follows:

1. The functional group plays a significant role in the initial decomposition reaction. For FOX-7, the hydrogen transfer reactions between -NH_2_ groups and -NO_2_ groups initiate the thermal decomposition at high temperature. In contrast, for FOX-7-T, the tetrazole ring breaks first at a much lower temperature to release N_2_, followed by numerous reactions.
2. The oxygen balance of the EM determines the composition of carbon aggregates during detonation, which greatly influences their detonation performance. The detonation products in the FOX-7 system are in the gas phase including very few simple carbon clusters, leading to a high energy release and a high external expansion capability. The reason is that most of the fuel atoms are accessible to oxygen to form gaseous products with no carbon cluster formation after adiabatic expansion. However, although FOX-7-T has a higher nitrogen percentage, its CJ pressure is 34.1% lower, leading to a 15.1% lower detonation velocity. This is because a much larger portion of the atoms in the FOX-7-T system were trapped into condensed phase carbon clusters at the CJ state, which remained even after the expansion of the reaction zone, suppressing the generation of gaseous products. The CJ temperature for FOX-7-T is predicted to be 45.4% lower than that of the FOX-7 system. Much less energy was released from the FOX-7-T system because many carbon atoms are not fully oxidized to form carbon clusters that trap many surrounding nitrogen atoms, suppressing the formation of N_2_ gases.

Our findings suggest that oxygen balance is an important factor to be considered in the design of the next generation of high-nitrogen-containing EMs. High nitrogen EM with a low oxygen balance like FOX-7-T can exhibit surprisingly low detonation performance because many and large carbon clusters form during detonation, suppressing the capability for external expansion. Moreover, a portion of the nitrogen atoms is trapped in the condensed clusters, further reducing the energy delivery.

## Supporting Information Available

Detonation products at the CJ State for FOX-7 with a C–O cutoff of 0.8 and 0.6 (PDF)

## References

[ref1] Sikder A. K.; Sikder N. A Review of Advanced High Performance, Insensitive and Thermally Stable Energetic Materials Emerging for Military and Space Applications. J. Hazard. Mater. 2004, 112 (1), 1–15. 10.1016/j.jhazmat.2004.04.003. 15225926

[ref2] Guo D.; An Q.; Goddard W. A. III; Zybin S. V.; Huang F. Compressive Shear Reactive Molecular Dynamics Studies Indicating that Cocrystals of TNT/CL-20 Decrease Sensitivity. J. Phys. Chem. C 2014, 118 (51), 30202–30208. 10.1021/jp5093527.

[ref3] Guo D.; An Q.; Zybin S. V.; Goddard W. A. III; Huang F.; Tang B. The Co-crystal of TNT/CL-20 Leads to Decreased Sensitivity Toward Thermal Decomposition from First Principles Based Reactive Molecular Dynamics. J. Mater. Chem. A 2015, 3 (10), 5409–5419. 10.1039/C4TA06858K.

[ref4] Boddu V. M.; Viswanath D. S.; Ghosh T. K.; Damavarapu R. 2,4,6-Triamino-1,3,5-trinitrobenzene (TATB) and TATB-Based Formulations-A Review. J. Hazard. Mater. 2010, 181 (1), 1–8. 10.1016/j.jhazmat.2010.04.120. 20554109

[ref5] Wang Y.; Liu Y.; Song S.; Yang Z.; Qi X.; Wang K.; Liu Y.; Zhang Q.; Tian Y. Accelerating the Discovery of Insensitive High-Energy-Density Materials by a Materials Genome Approach. Nat. Commun. 2018, 9 (1), 2444 10.1038/s41467-018-04897-z. 29934564 PMC6015015

[ref6] Winey J. M.; Toyoda Y.; Gupta Y. M. Near-Optimal Combination of High Performance and Insensitivity in a Shock Compressed High Explosive Single Crystal. J. Appl. Phys. 2021, 130 (1), 015902 10.1063/5.0057760.

[ref7] Tarver C. M.; Breithaupt R. D.; Kury J. W. Detonation Waves in Pentaerythritol Tetranitrate. J. Appl. Phys. 1997, 81 (11), 7193–7202. 10.1063/1.365318.

[ref8] Chapman D. L. VI On the Rate of Explosion in Gases. London, Edinburgh, and Dublin Philosophical Magazine and Journal of Science 1899, 47 (284), 90–104. 10.1080/14786449908621243.

[ref9] Davis W. C. The Detonation of Explosives. Sci. Am. 1987, 256 (5), 106–113. 10.1038/scientificamerican0587-106.

[ref10] Rabie R. L.; Fowles G. R.; Fickett W. The Polymorphic Detonation. Phys. Fluids 1979, 22 (3), 422–435. 10.1063/1.862610.

[ref11] Voelkel S. J.; Anderson E. K.; Short M.; Chiquete C.; Jackson S. I. Effect of Lot Microstructure Variations on Detonation Performance of the Triaminotrinitrobenzene (TATB)-Based Insensitive High Explosive PBX 9502. Combust. Flame 2022, 246, 112373 10.1016/j.combustflame.2022.112373.

[ref12] Shu J.; Pei H.; Huang W.; Zhang X.; Zheng X. Accurate Measurements of Detonation Pressure and Detonation Reaction Zones of Several Commonly-Used Explosives. Explos. Shock Waves 2022, 42 (5), 16–25.

[ref13] Winey J. M.; Zimmerman K.; Dreger Z. A.; Gupta Y. M. Structural Transformation and Chemical Stability of a Shock-Compressed Insensitive High Explosive Single Crystal: Time-Resolved Raman Spectroscopy. J. Phys. Chem. A 2020, 124 (32), 6521–6527. 10.1021/acs.jpca.0c04862. 32786234

[ref14] Winey J. M.; Toyoda Y.; Gupta Y. M. Shock Compression Response of an Insensitive High Explosive Single Crystal: 1,1-Diamino-2,2-dinitroethene (FOX-7). J. Appl. Phys. 2020, 127 (15), 155901 10.1063/1.5140194.

[ref15] Hemmi N.; Dreger Z. A.; Gruzdkov Y. A.; Winey J. M.; Gupta Y. M. Raman Spectra of Shock Compressed Pentaerythritol Tetranitrate Single Crystals: Anisotropic Response. J. Phys. Chem. B 2006, 110 (42), 20948–20953. 10.1021/jp0680589. 17048912

[ref16] Patterson J. E.; Dreger Z. A.; Miao M.; Gupta Y. M. Shock Wave Induced Decomposition of RDX: Time-Resolved Spectroscopy. J. Phys. Chem. A 2008, 112 (32), 7374–7382. 10.1021/jp800827b. 18642891

[ref17] Samuels P.; Spangler K.; Iwaniuk D.; Cornell R.; Baker E. L.; Stiel L. I. Detonation Performance Analyses for Recent Energetic Molecules. AIP Conf. Proc. 2018, 1979 (1), 150033 10.1063/1.5044989.

[ref18] Guo D.; Zybin S. V.; Chafin A. P.; Goddard W. A. III Increasing Oxygen Balance Leads to Enhanced Performance in Environmentally Acceptable High-Energy Density Materials: Predictions from First-Principles Molecular Dynamics Simulations. ACS Appl. Mater. Interfaces 2022, 14 (4), 5257–5264. 10.1021/acsami.1c20600. 35040628

[ref19] Gao H.; Joo Y.-H.; Parrish D. A.; Vo T.; Shreeve J. N. M. 1-Amino-1-hydrazino-2,2-dinitroethene and Corresponding Salts: Synthesis, Characterization, and Thermolysis Studies. Chem. - Eur. J. 2011, 17 (16), 4613–4618. 10.1002/chem.201002858. 21416511

[ref20] Frumkin A. E.; Yudin N. V.; Suponitsky K. Y.; Sheremetev A. B. 1-Amino-1-hydroxyamino-2,2-dinitroethene: Novel Insights in Chemistry of FOX-7. Mendeleev Commun. 2018, 28 (2), 135–137. 10.1016/j.mencom.2018.03.007.

[ref21] Tang Y.; Huang W.; Imler G. H.; Parrish D. A.; Shreeve J. N. M. Enforced Planar FOX-7-like Molecules: A Strategy for Thermally Stable and Insensitive π-Conjugated Energetic Materials. J. Am. Chem. Soc. 2020, 142 (15), 7153–7160. 10.1021/jacs.0c01640. 32227996

[ref22] Wahler S.; Klapötke T. M. Research Output Software for Energetic Materials Based on Observational Modelling 2.1 (RoseBoom2.1©). Mater. Adv. 2022, 3 (21), 7976–7986. 10.1039/D2MA00502F.

[ref23] Kresse G.; Hafner J. Ab Initio Molecular Dynamics for Liquid Metals. Phys. Rev. B 1993, 47 (1), 558–561. 10.1103/PhysRevB.47.558. 10004490

[ref24] Kresse G.; Furthmüller J. Efficiency of Ab-Initio Total Energy Calculations for Metals and Semiconductors Using a Plane-Wave Basis Set. Comput. Mater. Sci. 1996, 6 (1), 15–50. 10.1016/0927-0256(96)00008-0.

[ref25] Paier J.; Hirschl R.; Marsman M.; Kresse G. The Perdew-Burke-Ernzerhof Exchange-Correlation Functional Applied to the G2–1 Test Set Using a Plane-Wave Basis Set. J. Chem. Phys. 2005, 122 (23), 234102 10.1063/1.1926272. 16008425

[ref26] Grimme S.; Ehrlich S.; Goerigk L. Effect of the Damping Function in Dispersion Corrected Density Functional Theory. J. Comput. Chem. 2011, 32 (7), 1456–1465. 10.1002/jcc.21759. 21370243

[ref27] Kresse G.; Joubert D. From Ultrasoft Pseudopotentials to the Projector Augmented-Wave Method. Phys. Rev. B 1999, 59 (3), 1758–1775. 10.1103/PhysRevB.59.1758.

[ref28] Blöchl P. E.; Jepsen O.; Andersen O. K. Improved Tetrahedron Method for Brillouin-Zone Integrations. Phys. Rev. B 1994, 49 (23), 16223–16233. 10.1103/PhysRevB.49.16223. 10010769

[ref29] Zhou T.; Zybin S. V.; Goddard W. A. III; Cheng T.; Naserifar S.; Jaramillo-Botero A.; Huang F. Predicted Detonation Properties at the Chapman-Jouguet State for Proposed Energetic Materials (MTO and MTO3N) from Combined ReaxFF and Quantum Mechanics Reactive Dynamics. Phys. Chem. Chem. Phys. 2018, 20 (6), 3953–3969. 10.1039/C7CP07321F. 29367992

[ref30] An Q.; Goddard W. A. III; Zybin S. V.; Jaramillo-Botero A.; Zhou T. Highly Shocked Polymer Bonded Explosives at a Nonplanar Interface: Hot-Spot Formation Leading to Detonation. J. Phys. Chem. C 2013, 117 (50), 26551–26561. 10.1021/jp404753v.

[ref31] Guo D.; Zybin S. V.; An Q.; Goddard W. A. III; Huang F. Prediction of the Chapman-Jouguet Chemical Equilibrium State in a Detonation Wave from First Principles Based Reactive Molecular Dynamics. Phys. Chem. Chem. Phys. 2016, 18 (3), 2015–2022. 10.1039/C5CP04516A. 26688211

[ref32] Bian C.; Zhang M.; Li C.; Zhou Z. 3-Nitro-1-(2*H*-tetrazol-5-yl)-1*H*-1,2,4-triazol-5-amine (HANTT) and Its Energetic Salts: Highly Thermally Stable Energetic Materials with Low Sensitivity. J. Mater. Chem. A 2015, 3 (1), 163–169. 10.1039/C4TA04107K.

[ref33] Zhang J.; Hooper J. P.; Zhang J.; Shreeve J. N. M. Well-balanced Energetic Cocrystals of H_5_IO_6_/HIO_3_ Achieved by a Small Acid-base Gap. Chem. - Eur. J. 2021, 405, 126623 10.1016/j.cej.2020.126623.

[ref34] Baum K.; Bigelow S. S.; Nguyen Nghi V.; Archibald T. G.; Gilardi R.; Flippen-Anderson J. L.; George C. Synthesis and Reactions of 1,1-Diiododinitroethylene. J. Org. Chem. 1992, 57 (1), 235–241. 10.1021/jo00027a042.

[ref35] Bemm U.; Östmark H. 1,1-Diamino-2,2-dinitroethylene: A Novel Energetic Material with Infinite Layers in Two Dimensions. Acta Crystallographica Section C-Crystal Structure Communications 1998, 54, 1997–1999. 10.1107/S0108270198007987.

[ref36] Evers J.; Klapötke T. M.; Mayer P.; Oehlinger G.; Welch J. α- and β-FOX-7, Polymorphs of a High Energy Density Material, Studied by X-ray Single Crystal and Powder Investigations in the Temperature Range from 200 to 423 K. Inorg. Chem. 2006, 45 (13), 4996–5007. 10.1021/ic052150m. 16780321

[ref37] Crawford M. J.; Evers J.; Göbel M.; Klapötke T. M.; Mayer P.; Oehlinger G.; Welch J. M. γ-FOX-7: Structure of a High Energy Density Material Immediately Prior to Decomposition. Propell. Explos. Pyrot. 2007, 32 (6), 478–495. 10.1002/prep.200700240.

[ref38] Gao H.; Shreeve J. N. M. Recent Progress in Taming FOX-7 (1,1-Diamino-2,2-dinitroethene). RSC Adv. 2016, 6 (61), 56271–56277. 10.1039/C6RA12412G.

[ref39] Banik S.; Kumar Yadav A.; Kumar P.; Ghule V. D.; Dharavath S. Unfolding the Chemistry of FOX-7: Unique Energetic Material and Precursor with Numerous Possibilities. Chem. Eng.J. 2022, 431, 133378 10.1016/j.cej.2021.133378.

[ref40] Liu Y.; Li F.; Sun H. Thermal Decomposition of FOX-7 Studied by Ab Initio Molecular Dynamics Simulations. In Guosen Yan: A Festschrift from Theoretical Chemistry Accounts; Guo H.; Xie D.; Yang W., Eds.; Springer Berlin Heidelberg: Berlin, Heidelberg, 2015; 165–175.

[ref41] Ye C.-C.; An Q.; Goddard W. A. III; Cheng T.; Zybin S.; Ju X.-H. Initial Decomposition Reactions of Bicyclo-HMX [BCHMX or cis-1,3,4,6-Tetranitrooctahydroimidazo-[4,5-*d*]imidazole] from Quantum Molecular Dynamics Simulations. J. Phys. Chem. C 2015, 119 (5), 2290–2296. 10.1021/jp510328d.

[ref42] An Q.; Liu W.-G.; Goddard W. A. III; Cheng T.; Zybin S. V.; Xiao H. Initial Steps of Thermal Decomposition of Dihydroxylammonium 5,5′-bistetrazole-1,1’-diolate Crystals from Quantum Mechanics. J. Phys. Chem. C 2014, 118 (46), 27175–27181. 10.1021/jp509582x.

[ref43] Guo D.; An Q. Thermal Stability and Detonation Properties of Potassium 4,4’-Bis(dinitromethyl)-3,3′-azofurazanate, an Environmentally Friendly Energetic Three-Dimensional Metal-Organic Framework. ACS Appl. Mater. Interfaces 2019, 11 (1), 1512–1519. 10.1021/acsami.8b19611. 30525412

[ref44] Ye C.-C.; An Q.; Cheng T.; Zybin S.; Naserifar S.; Ju X.-H.; Goddard W. A. III Reaction Mechanism from Quantum Molecular Dynamics for the Initial Thermal Decomposition of 2,4,6-Triamino-1,3,5-triazine-1,3,5-trioxide (MTO) and 2,4,6-Trinitro-1,3,5-triazine-1,3,5-trioxide (MTO3N). Promising Green Energetic Materials. J. Mater. Chem. A 2015, 3 (22), 12044–12050. 10.1039/C5TA02486B.

[ref45] Karlsson S.; Östmark H.; Eldsäter C.; Carlsson T.; Bergman H.; Wallin S.; Pettersson A. Detonation and Sensitivity Properties of FOX-7 and Formulations Containing FOX-7; FOI, Swedish Defence Research Agency, Grindsjöns Research Center, SE-147, 2002, 25.

[ref46] Wild R.; Teipel U. In Characterization and Explosives Properties of FOX 7, International Annual Conference-Fraunhofer Institut Fur Chemische Technologie; Fraunhofer-Institut fur Chemische Technologie: Berghausen, 1999, 2004, 69.

[ref47] Trzciński W. A.; Cudziło S.; Chyłek Z.; Szymańczyk L. Detonation Properties of 1,1-Diamino-2,2-dinitroethene (DADNE). J. Hazard. Mater. 2008, 157 (2), 605–612. 10.1016/j.jhazmat.2008.01.026. 18282659

[ref48] Trzciński W. A.; Cudziło S.; Chyłek Z.; Szymańczyk L. Detonation Properties and Thermal Behavior of FOX-7-Based Explosives. J. Energy Mater. 2013, 31 (1), 72–85. 10.1080/07370652.2011.611579.

