# Supplementary material for: Detonation Performance of Insensitive Nitrogen-Rich Nitroenamine Energetic Materials Predicted from First-Principles Reactive Molecular Dynamics Simulations
Source: JACS Au. 2024 Mar 21;4(4):1605–14. doi: 10.1021/jacsau.4c00069 (PMC11040668; doi:10.1021/jacsau.4c00069)
Supplement: Supplementary file 1 — au4c00069_si_001.pdf [file au4c00069_si_001.pdf]

Detonation Performance of Insensitive Nitrogen-rich Nitroenamine Energetic Materials Predicted from First-Principles Reactive Molecular Dynamics Simulations

Dezhou Guo[1], Yuanyuan Wei[1], Sergey V. Zybin[2], Yan Liu[1], Fenglei Huang[1], William A. Goddard III[2*]*

[1]*State Key Laboratory of Explosion Science and Technology, Beijing Institute of Technology, Beijing 100081, People's Republic of China*

[2]*Materials and Process Simulation Center, California Institute of Technology, Pasadena, California 91125, United States*

*Corresponding author: huangfl@bit.edu.cn, wag@caltech.edu

The pressure in lammps program is calculated by

$$P = \frac{Nk_B}{V}T + \frac{1}{Vd}\sum_{i=1}^{N'} \vec{r_i} \cdot \vec{f_i} \quad (1)$$

where N is the number of atoms in the system, k_B is the Boltzmann constant, T is the temperature, d is the dimensionality of the system, and V is the system volume. $\vec{r_i}$ and $\vec{f_i}$ are the position and force vector of atom i.

Also, the kinetic energy in lammps program is calculated by

$$E_{kin} = \sum_{i=1}^{N_{atoms}} \frac{1}{2} m_i v_i^2 \quad (2)$$

where m_i and v_i are the mass and velocity of atom i.

Table S1. Detonation Products at the CJ State for FOX-7 with C-O cutoff of 0.8 and 0.6

		FOX-7	
		C-O (0.8)	C-O (0.6)
Main Products (mol/mol)	N_2	1.50±0.00	1.50±0.00
	CO_2	0.125±0.03	0.125±0.03
	H^+	0.55±0.11	0.55±0.11
	H_2O	0.125±0.04	0.15±0.04
	HO^-	0.08±0.03	0.11±0.04
	NH_3	0.25±0.00	0.25±0.00
	CHO_2	0.11±0.03	0.11±0.03
Other molecules (mol/mol)			
carbon clusters		0.61	0.72
composition		$C_{2.89}H_{3.30}N_{1.23}O_{5.46}$	$C_{2.61}H_{2.70}N_{1.04}O_{4.83}$
